# The Role of IgG4 in the Fine Tuning of Tolerance in IgE-Mediated Allergy and Cancer

**DOI:** 10.3390/ijms21145017

**Published:** 2020-07-16

**Authors:** Rodolfo Bianchini, Sophia N. Karagiannis, Galateja Jordakieva, Erika Jensen-Jarolim

**Affiliations:** 1 The Interuniversity Messerli Research Institute of the University of Veterinary Medicine, Medical University of Vienna and University of Vienna, Veterinaerplatz 1, 1210 Vienna, Austria; Rodolfo.Bianchini@vetmeduni.ac.at; 2 Institute Pathophysiology and Allergy Research, Center for Pathophysiology, Infectiology and Immunology, Division of Comparative Immunology and Oncology, Medical University of Vienna, Währinger Gürtel 18-20, 1090 Vienna, Austria; 3 St. John’s Institute of Dermatology, School of Basic & Medical Biosciences, King’s College London, 9th Floor, Tower Wing, Guy’s Hospital, London SE1 9RT, UK; sophia.karagiannis@kcl.ac.uk; 4 Breast Cancer Now Research Unit, School of Cancer & Pharmaceutical Sciences, King’s College London, Guy’s Cancer Centre, London SE1 9RT, UK; 5 Department of Physical Medicine, Rehabilitation and Occupational Medicine, Medical University of Vienna, Währinger Gürtel 18-20, 1090 Vienna, Austria; galateja.jordakieva@meduniwien.ac.at

**Keywords:** allergy, cancer, IgG4, immunotolerance, M2b-like macrophages, CCL1-CCR8 Tregs, regulatory cells

## Abstract

Among the four immunoglobulin G (IgG) subclasses, IgG4 is the least represented in serum of a healthy human and it is considered an “odd” antibody. The IgG4 antibody has unique structural features that affect its biological function. These include the ability to undergo antigen-binding fragment (Fab)-arm exchange, to create fragment crystallizable (Fc) – Fc binding with other IgG4 and other IgG subclass antibodies, have a unique affinity profile for Fc gamma receptors (FcγRs) and no binding to complement component C1q. Altogether, these characteristics support anti-inflammatory roles of IgG4 leading to immune tolerance. Under conditions of chronic antigenic stimulation and Th2-type inflammation, both tissue and serum IgG4 levels are increased. This review seeks to highlight how in allergen immunotherapy IgG4 can confer a protective role as a “blocking” antibody and safeguard from subsequent allergen exposure, while IgG4 can confer immunomodulatory functions to support malignancy. While Th2 conditions drive polarization of macrophages to the M2a subtype, chronic antigen stimulation drives B cell class switching to IgG4 to further support phenotypical macrophage changes towards an M2b-like state. M2b-like macrophages can secrete chemokine (C-C motif) ligand 1 (CCL1) and interleukin-10 (IL-10) to support regulatory cell recruitment and to further shape a tolerogenic microenvironment. Thereby, IgG4 have a Janus-faced role, favorable in allergy but detrimental in cancer.

## 1. Introduction

### 1.1. IgG Structures

The four human immunoglobulin (IgG) subclasses, defined IgG1, IgG2, IgG3, and IgG4 following their descending order of abundance, were discovered in the 1960s following extensive studies using specific rabbit antisera against human myeloma IgG proteins. Despite an amino acid homology of over 90%, each subclass has a unique structural and functional profile in antigen binding, immune complex formation, complement activation, effector cell activation, serum half-life, and placental transport [1]. Upon antigen contact, IgG3 is often the first subclass to form, while later responses are dominated by IgG1. IgG4 is often the result of repeated or prolonged exposure to an antigen. However, direct class switch from IgM- to IgG4-expressing naive B cell is also possible [2]. Two identical heavy chains and two identical light chain subunits interconnected by intramolecular disulfide bonds form the IgG as heterotetrameric glycoproteins. There are four types of heavy chains (γ1, γ2, γ3, and γ4) with a size of about 50 kDa. They are composed of a variable N-terminal domain (VH) followed by three constant domains (CH1, CH2, and CH3). There are only two types of light chains (κ or λ) with a size of about 25 kDa, and they are composed of a variable N-terminal domain (VL) and a constant domain (CL). The light and heavy chains join with the VL–VH and CL–CH1 domains to form two “Fab arms” that bind the antigen [2]. The region comprising the CH2 and CH3 domains is defined as fragment crystallizable (Fc). This region is responsible for the effector function, while the Fab binds antigens through the variable domains. A flexible hinge region, between the CH1 and CH2 domains, connects the Fab to the Fc region [3,4]. 

### 1.2. IgG and Fc Receptors

Antibody effector functions depend on the Fc domain interaction with effector molecules, comprising Fc gamma receptors (FcγRs), two members of the Fc receptor-like (FcRL) family (FcRL4 and FcRL5), complement components (C1q), the neonatal Fc receptor (FcRn), and tripartite motif-containing protein 21 (TRIM21) [4]. The FcγRs can be classified depending on their affinity to IgG: (1) one high-affinity receptor FcγRI (CD64), with the ability to bind monomeric IgG; (2) two low-affinity IgG receptors, FcγRII (CD32) with its sub-forms FcγRIIa, FcγRIIb, and FcγRIIc, and FcγRIII (CD16) with the sub-forms FcγRIIIa and FcγRIIIb—both low-affinity IgG receptors able to bind IgG-containing immune complexes. Each of these receptors can bind each of the four IgG isotypes with distinct affinity. 

The expression pattern of FcγRs is highly variable between different immune cells. For example, natural killer (NK) cells express only FcγRIIIa, while macrophages and monocytes express multiple receptors (FcγRIa, IIa, IIb, and IIIa) [4]. Another additional characteristic, that can further discriminate the FcγRs, is activatory versus inhibitory receptor functions. All FcγRs have activatory properties associated with an immunoreceptor tyrosine-based activation motif (ITAM) within cytoplasmatic domains, a conserved signal motif with the consensus sequence YxxI/ Lx_(6–12)_YxxI/L. The FcγRs associated with ITAMs either directly express the motif in their cytoplasmic tail (FcγRIIa and FcγRIIc-ORF) or are associated with a FcRγ-chain (FcγRIa and FcγRIIIa) expressing the ITAM motif. The activating FcγRs mediate effector function such as antibody-dependent cellular cytotoxicity (ADCC) and antibody-dependent cellular phagocytosis (ADCP) and the secretion of inflammatory mediators.

In contrast, the only inhibitory IgG receptor is FcγRIIb, associated with an immune receptor tyrosine-based inhibition motif (ITIM) within its cytoplasmic tail [3,4,5,6]. ITIM is a conserved signal motif with the consensus sequence I/V/L/SxYxxL/V, the x in both motifs representing any amino acid [6]. The simultaneous co-ligation of FcγRIIb with activating receptors can modulate immune responses [7]. The function of FcγRIIIb, the only glycosylphosphatidylinositol (GPI)-linked IgG receptor, is still uncertain [3].

**Figure 1:**
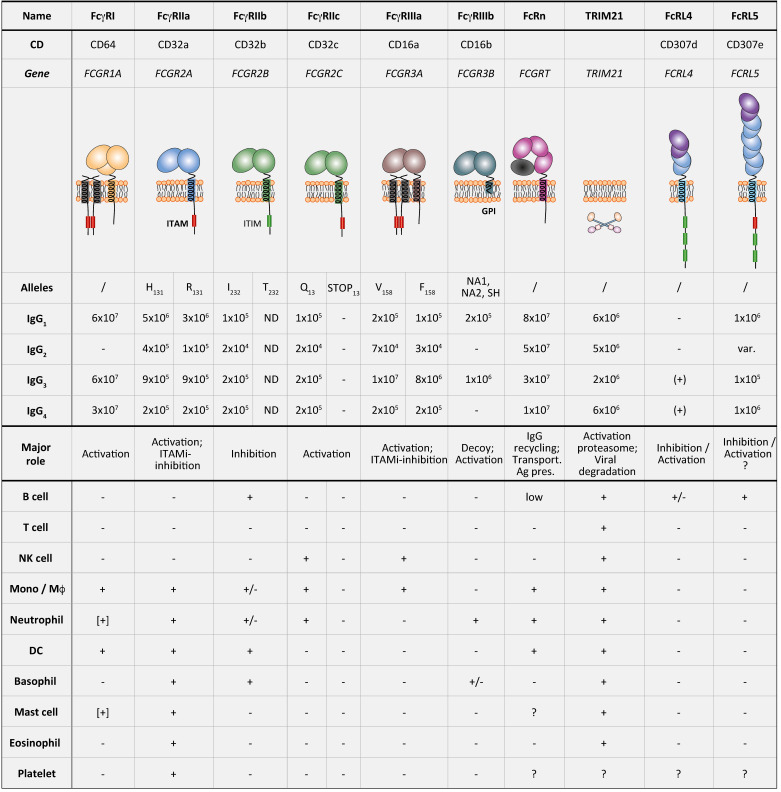
Fc gamma receptors (FcγRs) expression on immune cells. Re-adapted from Reference [8]. ITAM, immunoreceptor tyrosine-based activation motif; ITIM, immunoreceptor tyrosine-based inhibitory motif; GPI, glycosylphosphatidylinositol anchor. *Upper part*: binding affinities are indicated as Ka(M^−1^); −, no binding; (+), to be confirmed; ND, not determined; var., variable. *Lower part*: Expression patterns of FcRs are summarized as follows: +, expression; +/−, expression on a subpopulation; [+], inducible expression; ?, unknown.

FcγRs are expressed by different innate immune cells but also by some non-hematopoietic cells (Figure 1) [6]. FcγRI is constitutively expressed by monocytes/macrophages and some dendritic cells (DCs), and its expression can be induced on neutrophils, eosinophils, and mast cells mainly by IFN-γ stimulation and, to a lesser extent, by granulocyte colony-stimulating factor (G-CSF), interferon-α (IFN-α), and interleukin-12 (IL-12) [6,8,9]. FcγRIIa is expressed by macrophages, neutrophils, mast cells, eosinophils, and platelets. FcγRIIa is constitutively expressed by all myeloid cells, such as macrophages, neutrophils, mast cells, eosinophils, and platelets, but is absent on lymphocytes. The inhibitory FcγRIIb is expressed on B cells, basophils, tissue macrophages, DCs and on a small fraction of monocytes and neutrophils but not on mast cells. FcγRIIc is only expressed by individuals carrying the FCGR2C-ORF polymorphism (20–25% of the population) on NK cells, monocytes, and neutrophils. FcγRIIIa is expressed by NK cells and to a lesser extent by monocytes, macrophages, basophils, and mast cells. FcγRIIIb is expressed only by neutrophils and at low level by a subset of basophils [6,8,9]. It is important to note that the expression of FcγRs can be modulated by several factors. In fact, interferon-γ (IFN-γ), a Th1-type cytokine, upregulates the expression of activating FcγRs and downregulates inhibitory FcγRIIb. On the contrary, interleukin-4 (IL-4), interleukin-10 (IL-10), and transforming growth factor beta (TGF-β), all Th2-type cytokines, upregulate the expression of the inhibitory FcγRIIb [10,11].

### 1.3. IgG Production by B Cells and Relative Class Switch

To produce antibodies that maintain the antigen specificity but have a different effector function, proliferating B cells are subjected to class switch recombination (CSR). Class switch recombination is an intrachromosomal DNA recombination between the switch (S) region located downstream the variable diversity joining segments (VDJ) region, or variable heavy (VH) region, and the S region upstream the constant heavy (CH) region. The activation-induced cytidine deaminase (AID) enzyme controls this process [12]. In addition, T cells in the lymph node germinal centers direct the specific class switch by shaping the specific cytokine milieu in the B cell microenvironment. Several studies discovered that both IgG4 and IgE isotype switching are promoted by B and T cell interaction through CD40:CD40-ligand and by Th2-type cytokines (IL-4, IL-13) [3,13]. 

## 2. IgG4 Structure Characteristics and Its Specificity for Fc Gamma Receptors 

### 2.1. IgG4 Structure

IgG4 is the least abundant IgG subclass in human serum with an approximately total IgG relative abundance of 4–5% [1]. The structure and biological functions of IgG4 are less well understood than the more extensively characterized properties of IgG1 [7]. Aalberse et al. [14] have termed IgG4 as an “odd” antibody due to the fact of its unique biological properties and even considered this subclass to “break the rules” by not complying with the conventional understanding of antibody structure [14]. IgG4 contains unique structural features in the hinge region, CH2 and CH3 domains, that are thought to be responsible for its structural properties, binding characteristics, and reduced effector function, compared to other IgG subclasses. The structure of IgG4, in comparison with IgG, shows a shorter hinge region (12 amino acids) and a lower Fab arm flexibility [2,3]. The hinge flexibility influences antigen binding capacity, immune complex formation, and the binding sites for C1q and/or FcγR which can be partially or completely hidden by Fab arms [2,3]. Another region, considered critical for IgG binding to FcγRs and C1q is the FG loop (loop between strand F and strand G) of the CH2 domain [7]. High-resolution crystal structures of human IgG4-Fc revealed that the FG loop in the IgG4 CH2 domain adopts a conformation that disrupts the C1q and FcγR binding sites [7]. Together, these characteristics may be responsible for the poor ability of IgG4 to trigger effector functions by reduced engagement of FcγRs and C1q.

### 2.2. Fab Arm Exchange

**Figure 2:**
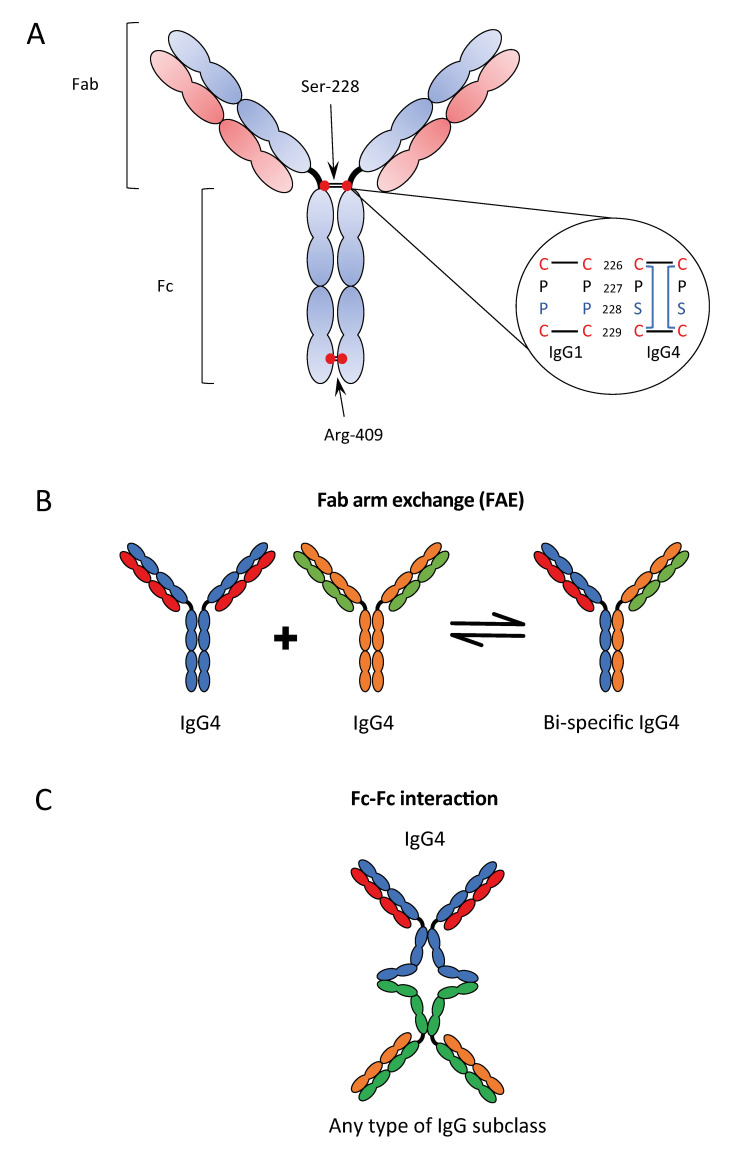
Characteristics of IgG4: structure of IgG4 antibody (**A**); Fab-arm exchange (**B**); and Fc–Fc interaction (**C**). Re-adapted from References [3,15].

An even more peculiar characteristic of IgG4 is the so-called Fab arm exchange (FAE); the exchange of two half-molecules, consisting of one heavy and one light chain (Fab arm) derived from two distinct IgG4 antibodies. Fab arm exchange enables the association of two different specificities within one IgG4 molecule, enabling a bi-specific configuration. The FAE process is facilitated by two unique features which weaken the interactions between the two Fab arms of the cognate IgG4 molecule: (1) the serine at position 228 in the core hinge region causing a cysteine–proline–serine–cysteine (CPSC) motif instead of the cysteine–proline–proline–cysteine (CPPC) sequence of the other IgG isotypes (Figure 2A); (2) the arginine at position 409 instead of lysine 409 in any other IgGs in the CH3 domain, causing a weak non-covalent interaction between the CH3 domains of two Fab arms, due to the disruption of the water-mediated hydrogen bonds at the CH3–CH3 inter-domain [4] (Figure 2B).

These features of IgG4 result in a more flexible core hinge domain and allow two hinge isomers: one with the typical inter-heavy chain disulfide bonds (covalently linked half molecules) and the other with intra-heavy chain disulfide bonds (non-covalently linked half molecules) [16].

Several studies determined that the FAE process is controlled by redox conditions and can be accomplished in vivo as well as in vitro by choosing an appropriate redox buffer. However, with a redox buffer that mimics plasma conditions, the IgG4 FAE rate was an order of magnitude lower than the actual rate of exchange calculated based on in vivo observations [16]. The exact way in which FAE exchange is controlled in vivo therefore remains elusive. 

A direct consequence of FAE is the phenomenon of functionally monovalent but bi-specific IgG4 antibodies that are unable to crosslink identical antigens or form large ICs against a specific target, yet even prevent formation of large immune complexes by other antibodies. In the last years, different therapeutic IgG4 antibodies were designed for treating several diseases, and a S228P mutation, which renders the IgG4 core hinge more IgG1-like, was considered to abolish FAE in vitro and in vivo [16].

### 2.3. Fc–Fc Interactions

Among the different unique characteristics of IgG4 there is also the Fc–Fc interactions that describe the interaction between human IgG4 and several other IgG subclasses, but not with other human Ig classes [17] (Figure 2C). This Fc–Fc binding was originally observed in the context of rheumatoid arthritis. It was demonstrated that the structural features important for FAE also control the Fc binding activity, the hinge isomers of IgG4, the intra-chain (non-covalently linked) form, and, in particular, the arginine-409 in the CH3–CH3 interface [17]. The reaction does not happen in suspension but requires one of the two antibodies to be bound in a solid phase, for example, to an antigen on a solid structure. The findings of IgG4 deposition in several autoimmune diseases called IgG4-related diseases (IgG4-RD) have been correlated with this characteristic to bind to deposited IgGs, but this remains unproven. Moreover, since Fc–Fc binding may be possible with other IgG subclasses suggests that IgG4 can act as a scavenger to IgG molecules with impaired structural integrity [17].

### 2.4. Affinity of IgG4 for FcγRs

In recent years, several studies demonstrated that there is a range of binding affinities for FcγRs by the four different IgG subclasses (Figure 1). The comparison between IgG4 and IgG1 subclass shows that the affinity of IgG4 for FcγRI is of the same order of magnitude as that of IgG1, while it is lower for FcγRIIa (Arg/His131) and FcγRIIIa (Phe/Val158) in comparison to that calculated for IgG1. The affinity of IgG4 for the inhibitory FcγRIIb is similar or even higher than any other IgG subclasses. There is no binding affinity reported for IgG4 or IgG2 for the FcγRIIIb [3,7,8]. 

### 2.5. Higher Antigen Affinities of IgG4 and Immune Functions Implications

It is known that often repeated or prolonged exposure to the antigen fosters IgG4 responses [18]. The Th2 cytokines IL-4 or IL-13 control B cell class switching to both IgG4 and IgE. However, in the “modified Th2 response”, the expression of the cytokines IL-10 [19], VEGF [20], IL-12 [21], and IL-21 [22], in the presence of IL-4, drive class switching to IgG4, without IgE production [3].

Naive B cells expressing IgM can directly switch to the IgG4 class [23]. Furthermore, a temporal model of IgE/IgG class switching proposes that naive B cells initiate a serial class-switch from IgM to IgG3, then to IgG1 and to IgG2, and finally to IgG4 [24]. In fact, in terms of genomic location of IGHC genes, the Cγ4 locus is the last of the IgG subclasses on chromosome 14 [2]. It is known that AID controls not only CSR but also somatic hypermutation (SHM) with a role in affinity maturation. In accordance, IgG4 can achieve a higher affinity compared to IgG1 or IgG3 for the same antigen. In short, IgG3 starts the early immune response followed by IgG1 with the major effect on antigen clearance. The subsequent switch to IgG2 slows down the inflammatory drive. In case there is then still a repeated or prolonged presence of antigen, class-switching to IgG4 occurs. The latter out competes any other IgG isotypes by higher affinity to the antigen and can stop the inflammatory response of the IgG1/FcγR activation by high-affinity interaction with the only inhibitory IgG receptor, FcγRIIb [24,25,26].

### 2.6. High Levels of IgG4 Expression 

As outlined above, IgG4 serum levels increase, mostly following repeated or chronic exposure to an antigen [3,18]. On one side, elevated tissue and serum levels of IgG4 are associated with chronic inflammation in several pathological conditions, such as rheumatoid arthritis, IgG4-related diseases (IgG4-RD), and pemphigus vulgaris. On the other side, elevated levels of IgG4 are also associated with immune tolerance. In fact, antigen exposure can also induce specific immune tolerance, for instance to bee venom after allergen-specific immunotherapy (AIT) in beekeepers [2,3,7,27]. However, elevated IgG4 is also associated with immune escape in helminthic and filarial parasitosis [28,29], and is observed in malignancies, such as melanoma [30], extrahepatic cholangiocarcinoma [31], pancreatic cancer [32], gastric cancer [33], colorectal cancer [34], and glioblastoma [35], potentially associated with unfavorable prognosis [3,7].

## 3. IgG4 as a Key Sign of Immune Tolerance in Allergen Immunotherapy 

Type I hypersensitivity (IgE-mediated hypersensitivity) is characterized by 2 phases: (1) the sensitization phase characterized by differentiation and clonal expansion of CD4^+^ producing Th2 cytokines such as IL-4 and IL-13 supporting isotype switch by B lymphocytes to allergen-specific IgE and subsequent binding of IgE to its high-affinity receptor FcεRI on mast cells and basophils; (2) the effector phase characterized by a subsequent encounter with the same allergen that crosslinks the IgE–FcεRI complexes on sensitized basophils and mast cells, leading to the release of anaphylactogenic mediators (effector phase) [27]. Importantly, the strong Th2 cytokine microenvironment also primes innate immune cells such as macrophages. The signals from the surrounding Th2 environment polarized macrophages into different functional phenotypes of alternatively activated anti-inflammatory M2 macrophages: M2a, M2b or M2c. The pro-allergic M2a phenotype is triggered by IL-4 and IL-13. The M2b and M2c phenotypes are involved in immune regulation, tissue remodeling, angiogenesis, and tumor progression [36]. 

After AIT, the levels of IgG4 in sera may rise 10–100 fold [27], and their appearance coincides with the development of immune tolerance [2]. IgG4 in AIT has been associated with a so-called “blocking function”, being able to prevent immediate hypersensitivity symptoms. Blocking mechanisms exploit all typical IgG4 characteristics: (1) high affinity to the allergen as a result of the affinity maturation process, thereby effectively trapping the allergen; (2) its ability for FAE resulting in bi-specific antibody that cannot be crosslinked easily; (3) the lack of binding pro-inflammatory complement; (4) high affinity for the inhibitory FcγRIIb expressed on effector cells and antigen presenting cells (APCs) [37]; (5) inhibition of IgE-facilitated allergen presentation (IgE-FAP) by direct competition with the allergen binding to IgE-CD23 complexes high affinity [38]. Together, these lead to reduced activation of memory IgE^+^ B cells by allergens and decreased IgE production. All five mechanisms synergize to counteract the allergic inflammation and support specific immune tolerance to the specific allergen. 

On the other hand, improvement of clinical symptoms throughout AIT, often correlates with increased production of IL-10 which is simultaneously responsible for both total and allergen-specific IgE suppression as well as IgG4 induction, the reduction of the IgE/IgG4 ratio and skewing towards a tolerogenic microenvironment. Regulatory T (Treg) cells are essential for the induction and maintenance of peripheral tolerance by preventing excessive immune responses. There are two types of human Tregs, distinguishing the naturally occurring thymus derived CD4+CD25+FOXP3+ Tregs, also called nTregs, and the inducible Treg cells (iTregs). Among the iTregs, other sub-categories: FOXP3 expressing iTregs, IL-10 secreting iTregs (Tr1), and TGF-β expressing iTregs (Th3) can be identified. Human Tr1 cells suppress T effector cells by various means: (1) they express C-C Motif Chemokine Receptor (CCR)2, CCR4, and CCR8 to inhibit the migratory capacity of T effector cells; (2) they express inhibitory receptors such as cytotoxic T-lymphocyte-associated protein 4 (CTLA-4), programmed cell death protein 1 (PD-1); (3) and they secret inhibitory cytokines such as IL-10, IL-35, and TGF-β [39,40]. 

It was shown that IL-10 secreting Tregs can selectively enhance IgG4 versus IgG1 production by B cells, and that this requires cell–cell contact, later shown to be dependent on glucocorticoid-induced TNFR-related gene (GITR) and TGF-β in addition to IL-10 [41]. This effect was only observed in the presence of memory B cells, suggesting that it is rather an effect on proliferation, while the switch factor IL-4 is required to induce IgG4 production [42]. It is possible that the presence of IL-10 results in a growth advantage of IgG4 switched B cells. However, phenotypic analysis of IgG4 B cells did not show enhanced expression of the IL-10 Receptor on either protein or mRNA levels as compared with IgG1 B cells [43]. Lighaam et al. [43] have isolated and characterized circulating IgG4 memory B cells and observed decreased expression of several surface proteins, including complement receptor 1 (CR1 or CD35) and CR2 (CD21) and the increased expression of FcεRII (CD23) on IgG4 memory B cells, compared with IgG1 memory cells [43]. Although functional assays were not performed, this suggests different sensitivity of IgG4 memory cells for regulation by immune complexes, including IgE containing complexes. In a recent study, B cells specific for the major bee venom allergen phospholipase A (PLA) isolated from non-allergic beekeepers showed increased expression of IL-10 and IgG4. These characteristics were analogous to B cells from allergic patients receiving AIT. The study also demonstrated that human inducible regulatory B (Breg) cells secreting IL-10, were expressing IgG4 [2,44]. Among different regulatory B cells, Bregs secreting IL-10 are called Br1 and regulate excessive inflammation via the release of IL-10 which, in turn, induces the differentiation of Tregs and further suppresses immune responses. Human Br1 cells are characterized by high expression of CD25 and CD71 and low expression of CD73 on the cell surface, and (similarly to IL-1Ra + Bregs) by upregulation of CD25, programmed death-ligand 1 (PD-L1), suppressor of cytokine signaling 3 (SOCS3), and glycoprotein A [39]. IgG4 antibody titers drop after treatment with the monoclonal anti-CD20 antibody rituximab which depletes B cells but not plasma cells, whereas the other IgG subclasses appear unaffected [2]. 

It should also be considered that many other cell types, including monocytes, DCs, NK cells, macrophages, and mast cells, can produce IL-10 [39,45]. Several studies also point out that the cellular source of AIT-induced IL-10 production may depend on the exact treatment modalities. In fact, not only Treg cells and Breg cells increased IL-10 production after AIT, but also mucosal macrophages in patients receiving grass pollen AIT [46], and monocytes from peripheral blood mononuclear cells (PBMC) derived from patients receiving bee venom AIT [47]. All these studies suggest an important role of Tregs, Bregs, and other cellular subsets in orchestrating the development of an IgG4-antibody response via IL-10. 

## 4. IgG4 as a Key to Immune Tolerance in Cancer 

Numerous studies provide evidence that chronic inflammation increases the risk of cancer [48,49,50]. Chronic inflammation in combination with a strong tolerogenic microenvironment operated by tumor cells and tumor-infiltrating immune cells, promotes tumor progression, and supports metastatic spread. Some of these features may be: (1) formation of tertiary lymphoid structures, containing functional germinal centers where antigen-driven antibody responses may occur [51,52,53]; (2) infiltration by M2-type macrophages [54] and Tregs [55]; (3) modified Th2 microenvironment with expression of IL-10, IL-4, VEGF, and TGF-β [56]; and 4) detection of IgG4 antibodies and/or IgG4^+^ B cells [30,33,57], together with Tregs [56] and Bregs [58] in the tumor microenvironment (TME) [3]. 

In recent years, one area in the emerging field of AllergoOncology compares the characteristics of specific immune responses in allergy, AIT, and cancer immune tolerance [59,60,61]. Like in AIT, IgG4 formed in tumor and tumor stroma is associated with chronic antigen challenge and with the Th2 cytokines IL-4 and IL-13, in combination with IL-10, released by Treg cells and M2-like macrophages in particular [60] but also by the tumor cells themselves [62]. In contrast to the beneficial IgG4 and tolerance response in AIT that results in a reduction of allergic symptoms, elevated levels of IgG4 in malignancies, such as melanoma, extrahepatic cholangiocarcinoma and other tumors may be associated with inappropriate immune tolerance [3,60].

The elevated levels of tissue and serum IgG4 have been associated with poorer prognosis in biliary tract cancers [63], in gastric cancer [33], in colorectal cancer [34], and in malignant melanoma [20,30]. Moreover, IgG4 was found to positively correlate with numbers of Tregs and to negatively correlate with cytotoxic T lymphocytes, supporting its involvement of immune tolerance mechanisms in cancer. Treg cells, with the support of IgG4, can re-educate host immune responses, thereby escaping immune clearance. Currently, it is not entirely clear whether tumor-associated IgG4 antibodies are reactive to tumor antigens, but early discoveries in malignant melanoma and glioblastoma are suggestive of this [20,35]. 

A study on human melanoma xenograft models in mice demonstrated that anti-tumor specific IgG4 antibody impaired and counteracted anti-tumor specific IgG1, and had weak anti-tumoral effector functions in vivo [20]. Several characteristics of IgG4, specific or non-specific, might explain how IgG4 could support cancer immune evasion. Innate immune effector cells trigger antibody-dependent cellular phagocytosis (ADCP) and cytotoxicity (ADCC) via antibodies. Macrophages express FcγRIIa which is involved in ADCP [64], while NK cells express FcγRIIIa involved in ADCC [65]. Even if IgG4 and IgG1 are able to bind FcγRI with the same order of magnitude, IgG4 has a lower affinity for FcγRIIa or no affinity for FcγRIIIa in comparison with IgG1 [8]. Such discrepancy, compared to IgG1, could result in the lower potency of IgG4 to mediate ADCC [66] and ADCP. At the same time, IgG4 can bind the inhibitory FcγRIIb with higher affinity than any other IgG subclass [8]. FcγRIIb can modulate both innate (macrophage, mast cell, and basophil activation) and adaptive immunity (DC activation and antigen cross-presentation) and perform inhibitory effects only when other FcγRs are co-engaged. IgG4 may dampen FcγR immune activation by co-engaging FcγRIIb together with the engagement of any other FcγRs by antigen-specific IgG1, even at lower concentrations than IgG1 [2,3]. Furthermore, since IgG4 is not able to trigger complement-dependent cytotoxicity (CDC) [7], any tumor specific IgG4 antibody competing with tumor specific IgG1 antibody indirectly reduces IgG1-mediated CDC. 

In fact, high levels of tumor non-specific IgG4 antibodies could exert their blocking functions via Fc–Fc interaction with tumor specific IgG1 antibodies linked on solid antigen, if the IgG1 heavy chains is partially dissociated [2]. In fact, the proteases of the TME, such as matrix metalloproteases (MMPs), are known to support neoangiogenesis, tissue remodeling, and thus promotion of cancer cell metastasis. It is currently up to speculation if these enzymes could even partly dissociate or cleave tumor-specific IgG1 antibodies, rendering them available for IgG4 Fc–Fc interaction [67]. In a recent breast cancer study, the presence of cleaved IgG antibodies was found in the TME in tumor tissue extracts [68].

## 5. IgG4–M2a Macrophage Interaction—A New Proposed Mechanism for IgG4-Mediated Tolerance in Allergy and Cancer

In both AIT and cancer, a modified Th2 microenvironment based on IL-10 production by Tr1 and Br1 cells can lead to high levels of IgG4 expression by B cells and the establishment of immune tolerance. This is beneficial in AIT, reducing classical Th2 allergic responses, but can be detrimental in cancer. In both models, macrophages might have a key role in establishing and maintaining immune tolerance. Macrophages are the most abundant immune cells in the lung (e.g., in allergic asthma [69]) as well as in the tumor microenvironment in solid tumors [70]. The different functional phenotypes of macrophages depend on signals from the surrounding environment. Accordingly, it is possible to mirror the Th1/Th2 classification for macrophages: the classical pro-inflammatory M1 macrophages, mainly activated by IFNγ, LPS, and other TLR ligands versus the alternatively anti-inflammatory M2 macrophages (i.e., M2a, M2b, and M2c). M2a macrophages, triggered by IL-4 and IL-13, positively correlate with the severity of airway inflammation in allergic asthma [71]. M2c macrophages are induced by glucocorticoids, TGF-β, and IL-10 and support induction of Tregs [72], which in tumors correlates with disease progression and poor prognosis. Interestingly, M2b macrophages, induced by IgG immunoglobulin complexes and LPS, upregulate IL-10 and chemokine (C-C motif) ligand 1 (CCL1) expression and have been reported in the context of both, allergy as well as cancer [73]. CCL1 secretion is critical to maintain the M2b phenotype in mice and humans [74]. It is known that macrophages have a degree of plasticity, and it is interesting to consider how IgG4 could affect their polarization. When recently the subclass-specific effects on M1 macrophages were investigated, IgG4 inhibited IFNγ signaling via FcγRI, favoring an M2-like phenotype [75]. The results of this study could explain the correlation between elevated levels of IgG4 and poor prognosis in several cancer types. In fact, the presence of M1 macrophages in the tumor microenvironment has been associated with prolonged survival of cancer patients, while a low M1/M2a ratio was associated with poor prognosis in a variety of murine models and human malignancies [3,76]. 

**Figure 3:**
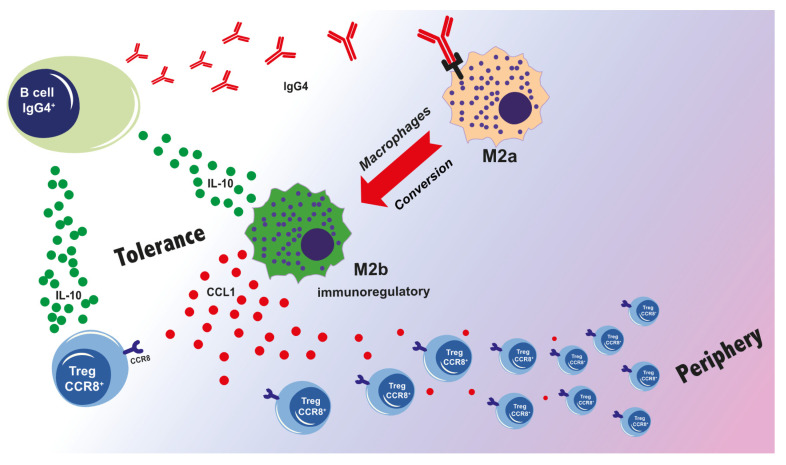
The loop of IgG4-induced immunotolerance [27,36]. Prolonged inflammation and chronic antigen stimulation result in an isotype switch to IgG4 which binds with high affinity to FcγRIIb on M2a macrophages and drives them towards an M2b type. The concurrent secretion of IL-10 and CCL1 is critical for Treg activation, for instance, via CCL1-CCR8 interaction. M2b and Tregs are an important source of IL-10 that, in turn, activates B cells, and further supports isotype switch to promote IgG4 production.

We have reported a phenotypic and functional change of pro-allergic M2a toward an M2b-like phenotype only in the presence of IgG4 but not IgG1 isotype antibodies [36]. Our data show that IgG4-mediated FcγRII stimulation is critical for the phenotypic and functional conversion of M2a to M2b-like macrophages. Even if IgG4 binds both FcγRIIa and FcγRIIb, more inhibitory FcγRIIb were expressed following IgG4 bindings to M2a macrophages [36]. The conversion of M2a into M2b-like macrophages could be inhibited by specific silencing of FcγRIIb (unpublished data). The M2b conversion can result in enhanced secretion of IL-10 and CCL1. While IL-10 further supports the class switch of B cells to IgG4-producing cells, CCL1 will mobilize CCR8^+^FOXP3^+^ Tregs from the periphery [56,77,78]. These observations emphasize the central role of IgG4 in establishing a tolerogenic microenvironment, prompting crosstalk between macrophages and Tregs (Figure 3).

## 6. Conclusions

IgG4 is the least abundant IgG subclass in human serum; nevertheless, it is unique among the IgG subclasses and presents distinct structural and functional features. The attributes of IgG4 comprise: (i) the ability to form FAE, a bi-specific antibody that is functionally monomeric; (ii) a lack of fixing complement and consequent inhibition of immune-complex formation by other isotypes; (iii) affinity for FcγRs, including the highest for inhibitory FcγRIIb; (iv) high antigen affinity that results from B cell affinity maturation during class switching. In consequence, IgG4 has a low capacity to trigger ADCP and ADCC and can antagonize IgG1 antibodies in cancer and IgE-mediated histamine release from allergic effector cells in AIT. IgG4 has also sustained immunosuppressive effects mediating a tolerogenic microenvironment by polarizing macrophages in immunoregulatory M2b-like cells, thereby presumably facilitating the recruiting of Tregs via CCL1-CCR8 interaction. Recently the CCL1-CCR8 axis was considered one of the most promising targets for tumor immunotherapy [55,56]. Due to the fact of its low immunoactivity properties, IgG4 is also considered a suitable subclass for the design of therapeutic antibodies for which effector functions are not desirable. Several therapeutic IgG4 antibodies, targeting the PD-1/PD-L1 axis and carrying the S228P mutation to enhance stability and inhibit FAE are available [56]. Future prospects for the use of the IgG4 isotype therapeutic antibodies should consider the critical structural and functional features of IgG4 in promoting immune tolerance. Several functional engineering studies to reduce the binding affinity of IgG4 isotype for FcγRs, especially for FcγRIIb, have been already done, but none were able to completely abolish the FcγRs–IgG4 binding [79,80]. It is probably worth re-thinking the value of therapeutic antibodies for cancer immunotherapy, considering their critical function in inducing immune tolerance being undesired in cancer.
